# Supplementary material for: IRIDA Phenotype in TMPRSS6 Monoallelic-Affected Patients: Toward a Better Understanding of the Pathophysiology
Source: Genes (Basel). 2022 Jul 23;13(8):1309. doi: 10.3390/genes13081309 (PMC9331965; doi:10.3390/genes13081309)
Supplement: Supplementary file 1 [file genes-13-01309-s001.zip › genes-1809945-supplementary.pdf]

Supplemental information

Methodology of MLPA [1]

All reagents for the MLPA reaction and subsequent PCR amplification were purchased from MRC-Holland (Amsterdam, The Netherlands), with exception of the *TMPRSS6* and control probes.

In short, in a one-tube format, combination of two adjacently annealing oligonucleotide probes were hybridized and ligated. After ligation, the common ends of the probes served as a template for PCR amplification with one primer pair and due to the fluorescent labeling of the primer the resulting products could be separated according to size using capillary electrophoresis on the ABI3130XL (Applied Biosystems). Fragment data were analyzed in GeneScan (Applied Biosystems). Peak heights of patient's samples were compared with control probes and ratios were calculated for all fragments (originating from *TMPRSS6* exons) using an Excel spreadsheet. Tresholds for deletions and duplications were set at 0.8 and 1.2 respectively, and all samples were tested at least twice.

Table S1. MLPA probes

target	Sequence L (5' > 3')	db nr	Length
TMPRSS6 ex1+2	gggttccctaagggttggaGTGAGCTGTCTCGGCACCCACTT	28324	42
IL2RB ex10	gggttccctaagggttggaGTTTCAGACCACAAGGGGCTCCACA	28322	44
TMPRSS6 ex7	gggttccctaagggttggaGCTCACAGGAATCAGGGGCTGTGCCCC	28332	46
TMPRSS6 ex11+12	gggttccctaagggttggaCCAGTCGGACCGTGAGTATGGGCAGCCGG	28338	48
TMPRSS6 ex3	gggttccctaagggttggaCCACTAACCCTGCTGGTTCCTTCCTGGGCAG	28326	50
KCTD17 ex8	gggttccctaagggttggaTACTGCCTGAGGGCCTTCAGGGACTTTGCTCCT	28348	52
TMPRSS6 ex13	gggttccctaagggttggaCCTCTCTGTGTCCCCACACAGTTTGCAGAGCCACA	28340	54
TMPRSS6 ex5+6	gggttccctaagggttggaGTGATCCTGGGTCAGTACTGCGAGTGGAAACGTGG	28330	54
TMPRSS6 ex16	gggttccctaagggttggaGGTAGAAAACCACCATTTAATGCGGCCTGTAGGCCCCTG	28344	58
TMPRSS6 ex8	gggttccctaagggttggaCCAGACCCCAGCCCAGCCTTGCCACCTTTC	28334	49
TMPRSS6 ex14+15	gggttccctaagggttggaCTCCTCCTCTGTCCACCCCACCACCTGCC	28342	48

TMPRSS6 ex4	gggttccctaagggttggaCCCAGGGGCTGTTGTCTTCCCTTCTCACAG	28328	49
TMPRSS6 ex9+10	gggttccctaagggttggaCCAGGGCCAGTGGACGATCCAGAACAGGAG	28548	49
TMPRSS6 ex17+18	gggttccctaagggttggaGAGAACAGGGGCTCCAGGCTCCTGAGATCTCA	28346	51

sequence R (5' > 3')	db nr	length
GCAGTCACTGCCGCCTGATGTTGtctagattggatcttgctggcac	28325	46
CCTTTGCTGTGTGTTCTGGGGCAACtctagattggatcttgctggcac	28323	48
TCTCCCCGCTCCAGGTTGTTACCGCTAtctagattggatcttgctggcac	28333	50
GGGAACCCCCTGCAGTGACTCGCTGCCTCtctagattggatcttgctggcac	28339	52
GGTACAAGGCGGAGGTGATGGTCAGCCAGGTtctagattggatcttgctggcac	28327	54
CTGTGCACCCTCACAACAACCCTGTGAGGTAGGtctagattggatcttgctggcac	28349	56
TTCCAGTGCAAAGAGGACAGCACATGCATCTCACTtctagattggatcttgctggcac	28341	58
GGTTGGCCTCATGAGGTTGGGGGAAACAAGCTGTGtgtatctagattggatcttgctggcac	28331	62
CCCCTCCCCTCCTAGCTCTTCCCTTCCTTCTGGAAGGGCtctagattggatcttgctggcac	28345	62
TGTTCTGCCAGGGTGTACGGCTGCAGCCGCggcgtatagggtgccgtggttgtctagattggatcttgctggcac	28335	75
CCCATCCCCAGACTGTGGCCTCCAGGGCCtaagtgggcgtatagggtgccgtggttgtctagattggatcttgctggcac	28343	80
CTCAAGGAGCTCATCACCAGCACCCGCCTGtgtaagtgggcgtatagggtgccgtggttgtctagattggatcttgctggcac	28329	83
GTACCACTTCCTCTCCTCCCTCTGGCTTCCtggttgtaagtgggcgtatagggtgccgtggttgtctagattggatcttgctggcac	28549	87
CTTCTGCCCTTGACCACGGACAGGCCCCATCAtggttgtaagtgggcgtatagggtgccgtggttgtctagattggatcttgctggcac	28347	89

Red: target left, green: target right, blue: Stuffer sequence, black: universal primer

Table S2. ***TMPRSS6*** **variants (predicted as nonpathogenic classes 1 and 2) found in patients**

Variants	ID														
**cDNA**	**12**	**1**	**13**	**15**	**5**	**9**	**16**	**2**	**4**	**8**	**6**	**14**	**10**	**7**	**11**
c.-120G>A	x			x	xx	xx	x	x	xx	x	x		xx	x	x
c.-113T>C	x			x	xx	xx	x	x	xx	x	x		xx	x	x
c.99G>A												xx			x
c.210G>A								x							
c.753C>T							x								
c.757A>G	x		xx		xx	xx		x	xx	x	xx	x	xx	x	x
c.1083G>A	x		x		xx	xx		x	x	x	xx	x	x	x	x
c.1254C>T				x			xx				x				
c.1563C>T	xx	x	x		xx	xx		x	xx	x	x	x	xx	xx	x
c.1654G>A														x	
c.1714G>A				x											
c.1869-6_1869-2del	x		x		x	x		x	x	x	x		x	x	x
c.2207T>C	xx	x	x		xx	xx		x	xx	x	x		xx	xx	x
c.2217C>T	xx		x		xx	xx		x	x	x	x		x	x	x
c.*503C>G			x												

cDNA: coding DNA. ID: patient ID (Table 1). 'x': heterozygous, 'xx': homozygous. In red: variants only found in patients.

Table S3. ***TMPRSS6*** **variants (predicted as nonpathogenic classes 1 and 2) found in relatives**

Variants	ID					
**cDNA**	**18**	**19**	**21**	**22**	**23**	**24**
c.-120G>A			xx	x	xx	x
c.-113T>C			xx	x	xx	x
c.99G>A						
c.210G>A						
c.753C>T						
c.757A>G	x	x	x	x	xx	x
c.1083G>A			x	x	xx	x
c.1254C>T				x		
c.1563C>T			xx	x	xx	x
c.1654G>A			x			
c.1714G>A						
c.1869-6_1869-2del			x	x	x	x
c.2207T>C			xx	x	xx	x
c.2217C>T				x	xx	x
c.*503C>G						

cDNA: coding DNA. ID: patient ID (Table 2). ‘x’: heterozygous, ‘xx’: homozygous

Table S4. Results of haplotype analysis

	Pathogenic variant *	c.-120G>A	c.-113T>C	c.757A>G	c.1083G>A	c.1563C>T	c.1869-6_1869-2del	c.2207T>C	c.2217C>T	Allele
ID 12 proband	2105G>T	x	x	x	x	x	x	x	x	M1
	Wt					x		x	x	P
ID 20 mother	2105G>T	x	x	x	x	x	x	x	x	M1
	Wt					x		x		M2

	Pathogenic variant *	c.-120G>A	c.-113T>C	c.757A>G	c.1083G>A	c.1563C>T	c.1654G>A	c.1869-6_1869-2del	c.2207T>C	c.2217C>T	c.*86C>T	Allele
ID 11 proband	c.1654G>A	x	x	x	x	x	x	x	x		x	A
	Wt					x			x	x		B
ID 21 sister	c.1654G>A	x	x	x	x	x	x	x	x		x	A
	Wt	x	x			x			x			C

	Pathogenic variant *	c.-120G>A	c.-113T>C	c.753C>T	c.757A>G	c.1083G>A	c.1254C>T	c.1563C>T	c.1869-6_1869-2del	c.2207T>C	c.2217C>T	Allele
ID 16 proband	c.1346A>G	x	x				x					M1
	Wt			x			x					P
ID 22 Mother	c,1346A>G	x	x				x					M1
	Wt				x	x		x	x	x	x	M2

#	*Pathogenic variant* *	c.-120G>A	c.-113T>C	c.210G>A	c.757A>G	c.1083G>A	c.1563C>T	c.1869-6_1869-2del	c.2207T>C	c.2217C>T	Allele
ID 2 proband	497delT	x	x		x	x	x	x	x	x	M1
	Wt			x							P
ID 3 mother	497delT	x	x		x	x	x	x	x	x	M1
	Wt	x			x		x		x		M2

	Pathogenic variant *	c.-120G>A	c.-113T>C	c.757A>G	c.1083G>A	c.1563C>T	c.1869-6_1869-2del	c.2207T>C	c.2217C>T	1699+20C>T	Allele
ID 8	c.863+1G>T	x	x	x	x	x	x	x	x		M1
proband	Wt										P2
ID 23	c.863+1G>T	x	x	x	x	x	x	x	x		M1
sister	Wt	x	x	x	x	x		x	x		P1
ID 24	c.863+1G>T	x	x	x	x	x	x	x	x		M1
mother	Wt									x	M2
ID n.a.	Wt									x	M2
brother	Wt	?	?	x	x	x		x	x		P1

*In grey: data from Supplemental Table 8, Donker et al.[2], * classes 3-5. Alleles M: maternal, P: paternal. # Proband and mother share a different wild type allele, but both express the IRIDA phenotype.*

Table S5. Overview of monoallelic IRIDA patients described in literature.

Study	Sex	Relation	Age (yrs)	Genotype	Hb (g/dL)	MCV (fl)	Ferritin (ug/L)	TSAT (%)	Hepcidin (ng/mL)	TSAT/hepcidin (%/nM)	Treatment
**Capra et al., 2017 [3]**	F	Proband	7	p.Arg446Trp; Wt	4.4	56	48	3	3.65	0.82	Red blood cell transfusion<br>IV iron<br>Liposomal iron
**Pellegrino et al., 2012 [4]**	F	Mother (1)	n.a.	IVS8-1433Δ9; Wt	7.5	65	3	2.2	n.a.	n.a.	Oral iron (poor response)
	F	Proband (2)	9	c.1869-21C>G (splicing); Wt #	10.6	69	19	4	2.5	1.6	Oral iron (poor response)<br>IV iron
	F	Mother (2)^	n.a.	c.1869-21C>G (splicing); Wt #	11.4	80	54	8	7	1.14	None
	F	Proband	17	p.His369Asn; Wt	8.1	79	116	4	n.a.	n.a.	IV iron
**Jaspers et al., 2013 [5]**	n.a.	Proband	52	c.1369+4A>T (splicing); Wt	10.6	n.a.	87 *	n.a.	n.a.	n.a.	IV iron
**Kannengiesser et al., 2009 [6]**	n.a.	Proband	13	c.335G>T; Wt (p.Arg112Leu)	n.a.	n.a.	40 *	n.a.	n.a.	n.a.	n.a.
	M	Proband	17 months	IVS16+1G>C; Wt (p.Gly713fs)	7.0	49	n.a.	5	n.a.	n.a.	n.a.
**Finberg et al., 2008 [7]**	M	Proband	7	c.2320C>T; Wt (p.Arg774Cys)	7.5	49	n.a.	4	n.a.	n.a.	n.a.
	F	Proband	5	p.Asp521Asn; Wt	10.3	70	20	n.a.	n.a.	n.a.	Oral iron
**Palare et al., 2010 [8]**	F	Mother	n.a.	p.Asp521Asn; Wt	n.a.	n.a.	n.a.	n.a.	n.a.	n.a.	n.a.

*Laboratory values at moment of diagnosis. In none of these patients CRP was determined. *Under iron suppletion,^ Suffering from an asymptomatic microcytic and hypochromic anemia, Proband (1) is not mentioned in this table since she is considered to have a compound heterozygous TMPRSS6 genotype and only mother (1) is considered as monoallelic IRIDA patient.*
# Proband and mother differ in single nucleotide polymorphisms (proband: rs11704654 and rs2235321; mother: rs4820268). (1) Family 1 in Pellegrino et al., (2) Family 2 in Pellegrino et al. TSAT: transferrin saturation, n.a.: not available.

1. White, S.J.; Breuning, M.H.; den Dunnen, J.T. Detecting copy number changes in genomic DNA: MAPH and MLPA. *Methods Cell Biol* **2004**, *75*, 751-768, doi:10.1016/s0091-679x(04)75032-3.
2. Donker, A.E.; Schaap, C.C.; Novotny, V.M.; Smeets, R.; Peters, T.M.; van den Heuvel, B.L.; Raphael, M.F.; Rijneveld, A.W.; Appel, I.M.; Vlot, A.J.; et al. Iron refractory iron deficiency anemia: a heterogeneous disease that is not always iron refractory. *Am J Hematol* **2016**, *91*, E482-e490, doi:10.1002/ajh.24561.

3. Capra, A.P.; Ferro, E.; Cannavò, L.; La Rosa, M.A.; Zirilli, G. A child with severe iron-deficiency anemia and a complex TMPRSS6 genotype. *Hematology* **2017**, *22*, 559-564, doi:10.1080/10245332.2017.1317990.
4. Pellegrino, R.M.; Coutinho, M.; D'Ascola, D.; Lopes, A.M.; Palmieri, A.; Carnuccio, F.; Costa, M.; Zecchina, G.; Saglio, G.; Costa, E.; et al. Two novel mutations in the tmprss6 gene associated with iron-refractory iron-deficiency anaemia (irida) and partial expression in the heterozygous form. *Br J Haematol* **2012**, *158*, 668-672, doi:10.1111/j.1365-2141.2012.09198.x.
5. Jaspers, A.; Caers, J.; Le Gac, G.; Ferec, C.; Beguin, Y.; Fillet, G. A novel mutation in the CUB sequence of matriptase-2 (TMPRSS6) is implicated in iron-resistant iron deficiency anaemia (IRIDA). *Br J Haematol* **2013**, *160*, 564-565, doi:10.1111/bjh.12147.
6. Caroline Kannengiesser, F.G., Laura Silvestri, Claire Oudin, Anne Marfaing, Leïla Chaiba-Berrouche, Jean Donadieu, Fabienne Toutain, Murielle Da ; Silva, B.I., Geneviève Marguerite, Patricia Aguila-Martinez, Clara Camaschella, Carole Beaumont and Bernard Grandchamp. Allelic heterogeinity of *TMPRSS6* mutations in IRIDA. **2009**.
7. Finberg, K.E.; Heeney, M.M.; Campagna, D.R.; Aydinok, Y.; Pearson, H.A.; Hartman, K.R.; Mayo, M.M.; Samuel, S.M.; Strouse, J.J.; Markianos, K.; et al. Mutations in TMPRSS6 cause iron-refractory iron deficiency anemia (IRIDA). *Nat Genet* **2008**, *40*, 569-571, doi:10.1038/ng.130.
8. Palare, M.; Ferrao, A.; Relvas, L.; Bento, C.; Morais, A. TMPRSS6 gene-Two new nonsense mutations associated with IRIDA. *Haematologica* **2010**, *95*, 704.
